# Use of Auditory Cues and Other Strategies as Sources of Spatial Information for People with Visual Impairment When Navigating Unfamiliar Environments

**DOI:** 10.3390/ijerph19063151

**Published:** 2022-03-08

**Authors:** Hisham E. Bilal Salih, Kazunori Takeda, Hideyuki Kobayashi, Toshibumi Kakizawa, Masayuki Kawamoto, Keiichi Zempo

**Affiliations:** 1 Graduate School of Comprehensive Human Sciences, University of Tsukuba, Tsukuba 305-8572, Japan; hishamelr@gmail.com; 2 Faculty of Human Sciences, University of Tsukuba, Tsukuba 305-8572, Japan; takedak@human.tsukuba.ac.jp (K.T.); hidekoba@human.tsukuba.ac.jp (H.K.); kakizawa@human.tsukuba.ac.jp (T.K.); 3 Headquarters for International Industry-University Collaboration, University of Tsukuba, Tsukuba 305-8550, Japan; m.kawamoto@farmcase.tech; 4 Faculty of Engineering, Information and Systems, University of Tsukuba, Tsukuba 305-8573, Japan

**Keywords:** natural sounds, auditory cues, mobility, visual impairment

## Abstract

This paper explores strategies that the visually impaired use to obtain information in unfamiliar environments. This paper also aims to determine how natural sounds that often exist in the environment or the auditory cues that are installed in various facilities as a source of guidance are prioritized and selected in different countries. The aim was to evaluate the utilization of natural sounds and auditory cues by users who are visually impaired during mobility. The data were collected by interviewing 60 individuals with visual impairments who offered their insights on the ways they use auditory cues. The data revealed a clear contrast in methods used to obtain information at unfamiliar locations and in the desire for the installation of auditory cues in different locations between those who use trains and those who use different transportation systems. The participants demonstrated a consensus on the need for devices that provide on-demand minimal auditory feedback. The paper discusses the suggestions offered by the interviewees and details their hopes for adjusted auditory cues. The study argues that auditory cues have high potential for improving the quality of life of people who are visually impaired by increasing their mobility range and independence level. Additionally, this study emphasizes the importance of a standardized design for auditory cues, which is a change desired by the interviewees. Standardization is expected to boost the efficiency of auditory cues in providing accurate information and assistance to individuals with visual impairment regardless of their geographical location. Regarding implications for practitioners, the study presents the need to design systems that provide minimal audio feedback to reduce the masking of natural sounds. The design of new auditory cues should utilize the already-existing imagination skills that people who have a visual impairment possess. For example, the pitch of the sound should change to indicate the direction of escalators and elevators and to distinguish the location of male and female toilets.

## 1. Introduction

The main hurdle that people with visual impairments need to clear to achieve independent living is the amount of information they can gain by interacting with an unfamiliar environment [1,2,3,4]. According to Jonnalagedda et al. [5], people with a severe visual impairment or blindness experience the world in a way that is different from those who are sighted. Researchers have argued that apart from the challenge of communicating through reading and writing, the inability to travel independently and to interact with their surroundings is the most significant problem that is faced by the visually impaired [6,7,8,9,10,11].

However, to keep the visually impaired up to date with necessary information about their environment in order to secure their safety and independence of mobility, this segment of the populations needs to learn special mobility skills. They also need to depend on non-visual information or other environmental information [12]. Various studies have shown that people who are visually impaired show higher levels of auditory ability due to their frequent use in this population. These abilities are pertinent to auditory skills such as echo processing, voice localizing, and distance estimation [13,14,15,16].

Sound, whether it is direct, indirect, or reflected, is the main source of information for the visually impaired. Ambient sound, regardless of whether it is natural or artificial, contains information about its source, including distance and, possibly, direction [17]. Humans use auditory senses to gain information from the environment, and this is also the case in the modern world through the use of various personal devices. Sound that emanates when pushing a button on an electric device or that is generated by a machine when it malfunctions are examples pointed out by Kurakata et al. [18]. They also list some of the most popular audible signals specifically designed for the visually impaired, including sounds from devices installed in modern hybrid cars (that warn people of their approach) and those from traffic signals at pedestrian crossings.

Our eyes help us to gather large amounts of information despite being limited directionally, while our ears are multidirectional and are able to gather information from a number of sources, as indicated by Seki [19]. Any audible sound has the potential of becoming an orientation aid; thus, the visually impaired should take advantage of this by becoming active listeners and should learn techniques to convert such cues into orientation aids, according to Hill and Ponder [20]. As stated by previous researchers [21,22,23,24,25], sounds, including announcements that repetitively emanate from beacons at shopping malls and train stations, for example, often confuse users who are not familiar with them, leading to a lack understanding and loss of meaning. However, auditory cues have great potential to compensate for vision loss via conveying visual information to the visually impaired to assist them in performing basic navigational tasks.

In this paper, auditory cues refer to auditory assistance for mobility aiming to support visually impaired people and that have mainly been installed in public passenger facilities, such as in railroad stations as defined by Ministry of Land, Infrastructure, Transport and Tourism (MLIT) of Japan (Ministry of Land, Infrastructure, Transport and Tourism, “Guidelines for Facilitating the Movement of Passengers in Public transport Facilities”, 2007). Auditory cues have become the most important means of providing information for the visually impaired in public spaces when it is difficult for them to obtain information from usual visual signs. Thus, various countries started to adapt sound guidance systems to provide much more support for users who are with visual impairment to be able to use public transport independently.

As reported by Sobnath et al. [26], the European Union has inaugurated a project identified as the European Innovation Partnership on Smart Cities and Communities (EIP-SCC). This project is aiming to establish, for smart cities that have the potentials of equipping the public facilities with mobile-assisted apps and other devices such as Audible and tactile signals for pedestrians, on board announcements on buses and trains, mobile apps that provide notifications near bus stops to be alerted when the intended bus is approaching, and product recognition system for more accessible shopping. Likewise, beacon-powered devices that can work both indoors and outdoors to send instant information to mobile devices via Bluetooth are being adapted as supplementary information source during mobility for the visually impaired in the city of Warsaw in Poland known as The (Virtual Warsaw) project, as reported in Košt’álová et al. [27] They also reported a combination of tactile paving block and an acoustic guiding beacon in the Czech Republic.

In Japan, audio-guidance systems for providing information for users who are visually impaired have been placed in many public places such as train stations and entrances of some public facilities in addition to acoustic traffic signals for assisting blind pedestrians. Likewise, devices for audio guidance systems also exist inside subway stations, which emit various sounds such as a combination of two-pitch tones to indicate entrances or ticket and bird chirping sounds to indicate stairs locations, as mentioned by Iwamiya et al. [28].

There are conspicuous legislative efforts to enhance the safety of users who are with visual impairment throughout their wayfinding, and various countries have enacted laws and legislations to regulate and facilitate the installation of auditory cues in various public facilities. In Japan, for instance, according to Inagaki [29] and Kose [30], The “Act on Promotion of Smooth Mobility for the Elderly and Persons with Disabilities (commonly known as the New Barrier-Free Act)” was enacted in December 2006. The act obliges public transport operators to comply with the barrier-free standards set forth in the act when constructing new or when upgrading passenger facilities such as railroad stations. This law also indicates a set of guidelines and certain rules for the installation of auditory cues to facilitate mobility in transportation facilities for users who are visually impaired. According to the Ministry of Land, Infrastructure, Transport and Tourism (MLIT), (2007) in July 2007, the “Guideline for the Development of Passenger Facilities for Public Transport to Facilitate Mobility” commonly known as the “Barrier-Free Development Guideline” was enacted. According to Bentzen and Mitchell [31], in the United States, earlier laws such as the Architectural Barriers Act (ABA) in 1968 directed all the architecture of the public facilities to be modified to accommodate the needs of the people with disabilities. As reported in Aldousari et al. [32] and Becerra [33], Americans With Disabilities Act 2010 ADA Standards instructed service providers to reasonably modify their policies in order to satisfy the law requirements when dealing with people with disabilities. These regulations have emphasized that gaining information via auditory cues or other assistive devices is not intended to replace the traditional mobility aids. It should rather be considered as a supplement source of information, and thus, the primary objective for enhancing gaining information via the auditory cues and other assistive devices is to help people with visual impairment improve their daily autonomy and ability to mentally represent their environment beyond what is possible with traditional assistive devices.

### 1.1. Significance of the Study

Although the limited sample size will make it more difficult to generalize the findings of the present research, this study will provide starting points toward enhancing upcoming auditory cues as a source of information for people who are visually impaired while mobile. Moreover, the backgrounds of the participants included in this study are of great value. In addition to exploring the ways audible signals affect the visually impaired as they go about their day and providing the suggestions and expectations raised by the participants about how adjustments to audible signals could boost their independence and help them maneuver their environments successfully, this study also investigates the strategies these participants use for wayfinding in their respective countries, which is of great importance since the impact of economical, cultural, and environmental elements manifest themselves in the different strategies that people use to acquire information from available auditory cues.

### 1.2. Research Questions

Since the varied backgrounds of participants in this study reflects a large variation in cultural and economic attitudes and behaviors during independent mobility, this paper proposes that an in-depth investigation into the already-existing auditory cue systems in various countries is important for the design of an auditory cues system that is a more reliable source of information than the already-existing country- or culture-specific ones. As such, this study raises the following questions:

Can additional enhancements in the design of auditory cues provide a supplemental beneficial information resource for users who are visually impaired in a given situation?

What are the enhancements that may be needed when designing new auditory cues so as to make them more recognizable from other existing sounds?

What are the priority facilities for installing auditory cues for the visually impaired?

### 1.3. Study Objective

This study aims to achieve the following objectives: to investigate the use of auditory cues that can be used by people with a visual impairment from different cultural backgrounds when navigating unfamiliar environments and to analyze the types of auditory cues that can be used indoors and outdoors and to investigate the effect of economical context on the prioritization of auditory cues and other strategies by the visually impaired.

## 2. Method and Participants

To collect sufficient, in-depth details on mobility habits among the visually impaired, the participants were given a set of open-ended questions through a semi-structured interview technique that allowed them to express their views freely. This method allows for the questions to be listed in a sequential order, but it also offers space for the interviewer to generate new questions depending on the participants’ answers. The interview questions were designed based on a previous study and the related literature. The designed interview questions underwent preliminary questions with three people who worked in the education field or who worked to provide services for the visually impaired. Their views and suggestions augmented the interview contents to guarantee that the questions that were asked covered all of the points that were intended to be investigated. This additionally ensures that the participants were given enough opportunity to freely express their thoughts.

Firstly, the participants were asked to provide their demographic information. Then, they were asked to offer information pertinent to the devices that they use for wayfinding; the next questions pertained to the strategies that they use to acquire information in unfamiliar locations; the next discussion session was intended to investigate how auditory cues can be helpful as a source of information in unfamiliar locations, and the participants were also requested to suggest locations where they felt auditory cues should be installed; finally, they were also asked to outline their hopes and any additional requests regarding sound elements that are easy to recognize as sources of information.

The interviews took between 30 and 60 min and were conducted through face-to-face sessions, phone calls, or video conference calls. The participants were recruited through international organizations that are a part of the interviewer’s professional network. A total of 60 participants from 14 countries agreed to participate. A total of 18 of them were from Japan, 12 were from Sudan, 5 were from South Korea, and there were 4 participants from each of the following countries: the United States of America, the United Kingdom, and Egypt. A total of 3 participants were from Kyrgyzstan and Thailand; 2 participants were from New Zealand and China, and there was 1 participant from each of the following countries: Myanmar, Jordan, and Indonesia.

All of the participants were blind or partially sighted and were older than 16 years of age. The data collected through these interviews were anonymized for confidentiality. The interviews were recorded after the participants verbally reaffirmed their consent for audio recording in compliance with ethical standards and research procedures of the University of Tsukuba in Japan. Table 1 provides more information regarding the profiles of the participants.

### Data Analyses

The interview questions were qualitatively analyzed by adapting the content analysis technique [34]. The interviews were audio recorded and later transcribed. The first author and colleague independently went through the interviews and created an initial set of codes based on the interview questions. Afterward, they met to discuss the similarities and differences in their initial set of codes and agreed on a codebook. Then, the codebook was used to code the interview data. When appropriate, a frequency count was also used to describe the number of responses within each category.

## 3. Results

In response to the questions concerning the necessary information the participants initially want to know when maneuvering in an unfamiliar location, the participants expressed a variety of views regarding necessary information that people who are blind or visually impaired need to know when traveling in unfamiliar environments. The variation in these views can be attributed to the fact that the participants use different means of transportation when traveling. There seems to be a very clear contrast in the responses between participants from developed cities who use trains and those who use other methods of transport from slightly developed cities, as seen in Table 2. We used The World Bank country data (The World Bank country data, https://data.worldbank.org/country, accessed on 25 February 2022) to categorize cities in countries with high and middle levels of income in a group comprising developed cities and cities located in countries with low- and middle-income levels, low-income levels, lower-middle-income levels as slightly developed cities.

In addition to expressing concerns about the physical layout of the stations, the participants said that they were concerned about safety and security. A male participant from Japan who was over 60 years of age and who worked in a facility that provided services for the visually impaired shared that *“There is a need for warning signs to alert people who are blind when approaching the platform’s edge. This is especially important since we heard of new reports about users who are blind [sometimes] stumbling or falling to the ground at the platform’s edge at Tokyo’s railway stations.”* Another Japanese male participant from Tokyo who was over 30 years of age and working a school teacher said that *“There is a need for detailed identification of different locations and directions for exits and stairs—particularly in large stations with multiple exits, stairs, and joint stations—since it is difficult for us to identify the exact exit or stairs leading to the desired destination.”* Similar results were reported in Goto et al. [35]; the authors emphasized the need for immediate sources of information—such as audible signals—since blind users cannot recognize the direction in which the stairs or escalators leading to the gates are moving. The same problem is experienced at doors when exiting a train.

In response to a question on the type of auditory cues used as a source of information in unfamiliar environments, the participants’ answers varied based on their preferred mode of transport. The users who primarily commuted via train relied on artificial sounds generated by different machines at train stations, while those who did not mainly depend on trains relied on natural ambient sounds, such as those mentioned in Table 3. This table revealed significant differences between the participants from different countries. For instance, the participants from developed countries mainly relied on designed auditory cues for the visually impaired, such as auditory cues emanating from beacon devices at train stations, cashiers, and the beep of electronic cards on buses, whereas participants from slightly developed cities offered a variety of natural sounds, including the sound of river water flow, animals, and birds.

One male participant from Egypt who described himself as being over 40 years old and unemployed shared his experience using natural sounds by saying *“I am always trying to get enough information concerning the surrounding environment, for the reason that I may need to refer to it in the future. It is vital for me to know what is around me, such as school, bank, drink shop, any brand company such as Macdonalds or KFC or other public facility such as cosmetics, gas station, etc. Also, I listen to every available ambient sound such as car repairing shop, coffee shops, and restaurant. Interestingly, various sounds can be limited to Egypt such as frying pan heating oil, coffee shop, people voices, and car speed bumps.”* Another schoolteacher from Sudan living in the capital city of Khartoum who was over 60 years of age shared the following: *“I usually check up the mean of transportation if it is taxi, bus, or on foot. I need to know any landmark that is near to my destination, such as a tree, famous building, or shop. I count the streets sometimes. Other landmarks are useful such as the slope and other hints that I can feel on the ground such as small stones and concrete. Music from various stores or cafeterias and footsteps are also a source of information I depend on in various occasions.”* Pre-existing natural audible sounds, such as the sound of scissors at a barber shop, people selling items on the street, mobile catering vehicles, deep-oil fryers, glass cups at coffee shops, the sound of wind at corners, cars passing over speed bumps, car maintenance equipment, and the sounds of people at various places such as kindergartens, schools, and playgrounds, are mutually used by participants from developed and developing countries.

**Figure 1:**
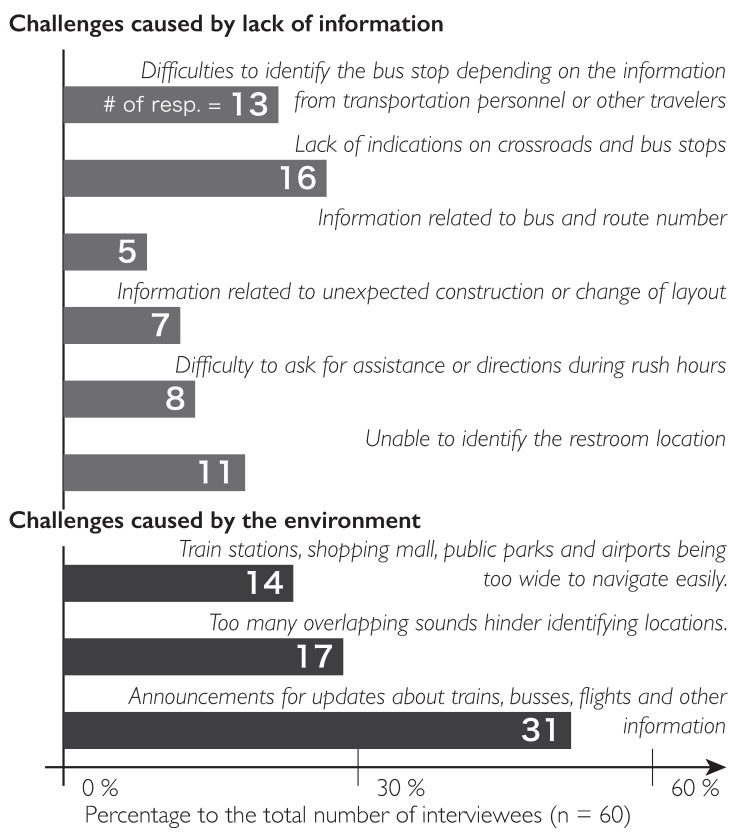
Basic challenges faced by visually impaired travelers.

The interview data also revealed one common concern and significant challenge among the participants when asked about the challenging process of gathering and maintaining updated and intensive information on the location being traversed. Figure 1 lists the answers of the participants concerning the basic challenges face by the visually impaired when traveling independently.

The participants were asked to express their views regarding the locations in which auditory cues should be installed. The participants’ views overwhelmingly exposed their hopes regarding the installation of auditory cues at different locations. The participants’ answers varied widely, reflecting differences between train users and those who use other modes of transportation, and these are mentioned in Table 4. As seen from this table, the train users pointed to a need to install auditory cues in railway station facilities, such as at the station entrance, and the need to distinguish between the upward- and downward-moving escalators. They also pointed to the need to install auditory cues for other facilities—such as to distinguish the male and female toilets and turnstiles, ticket offices, exits, and elevators. The importance of installing auditory cues at the areas mentioned above is also highlighted in Kurakata et al. [18].

A male participant from Seoul, South Korea in his late twenties and working as a school teacher expressed his view regarding the noticeable voices that can be used as a reliable source of information during mobility, saying, *“Using the already existing imaginative ability that people who are blind possess is extremely beneficial when designing new sounds. For example, the pitch should change to indicate the direction escalators and elevators are going”.* The data revealed that the type of information needed by the visually impaired differs based on gender and lifestyle. A female participant from Tokyo, Japan who was over 30 years of age said *“It is necessary to set priorities when installing audible cues in areas based on the basic personal needs of users. Those who enjoy walking may need public parks entrances to be marked with audible cues. If a female parent needs to pick up a child from kindergarten, as in my case, it would be convenient if the exit was equipped with an audible cues”.* In another case, a male participant from Tokyo, Japan who was over 50 years old and working in a library shared that, *“A problem many people who are blind face is the inability to distinguish between male and female toilets, and the in/out ticket gates at railway stations. Using the wrong gate leads to an unintentional collision with other users or may cause people who are blind to stumble on their canes. So, injuries may happen. The same situation applies to escalators, since it is impossible to distinguish between upward- and downward-moving escalators. These facilities need to be equipped with distinctive audible signals to prevent accidents”.* This statement reiterated a view stating that auditory cues should be related to the item that it intends to indicate. Therefore, users can take full advantage of using auditory cues, thus making it easier for pedestrians who are visually impaired to identify their distance through auditory cues.

When considering future assistive technology that works for most members of the visually impaired community, there seems to be a consensus on the need for devices that provide on-demand minimal auditory feedback and that transmit more information than can be achieved via the traditional white cane. These sentiments were expressed by a female government employee from the United states who was over 40 years of age: *“It will be extremely confusing when all the sounds I want to hear speak to me at the same time. I would rather prefer it if there were buttons or transmitters on my cellphone, for instance, that I can use to select what I hear. Devices with receivers should be set up to make a noise when users get very close to the entrance of their intended destinations. If I want to go to the pharmacy, the mailbox, or the bank, for instance, all I have to do is get the direction to reach my destination, since my cellphones have a language setting that allows me to choose a language I’m most comfortable with. When I get closer to my destination, there is no need for language guidance; an audible sound would be more useful for me. I can push the correct button repeatedly and follow the beeps until I get to the entrance”.* Regarding the use of new technology, the participants presented readiness to use new devices that provide information using minimum audible cues. They have affirmed that if they have to receive information from such devices, then they would prefer to do it manually by themselves through a transmitter, for instance, so they can receive the information when needed.

## 4. Discussion

A basic objective of this study was to shed light on the strategies that people who are visually impaired used when navigating an environment, including natural sounds and auditory cues, to acquire the necessary spatial awareness. Thus, the interview questions were designed to solicit basic information that seems to be crucial to those individuals before, during, and after traveling. However, in countries where there are no auditory cues designed for the visually impaired, the participants utilized available natural sounds. By translating these sounds into information, they are able to recognize the layout of the environment, identify and localize objects, maintain the correct direction, walk in a straight line, and avoid hazards. Similar results have been reported in previous research [17,36,37].

Train users encounter more problems than those who use other means of transport. In many cases, they have to rely on their own judgment to accomplish certain tasks, such as buying tickets, reading fares, and moving to a platform [38,39,40,41,42,43,44,45]. Such tasks require auditory cues based on the behavior of other users, and these may not be available when needed, as discussed in [23]. Other tasks, such as identifying ticket gates, entering a train, detecting the location of station entrances, and locating stairs and exits, can also be troublesome, as discussed by Goto et al. [35]. The stress that results from performing several tasks simultaneously is a primary hindrance that inhibits the implementation of independent mobility for the visually impaired. Using ticket vending machines or finding the locations of electronic card stands may become a complicated task, since these machines differ even within the same city, or depending on the manufacturing company. Installing large print instructions, Braille, audible signals on electronic card stands, and designating staff as helpers may offer practical solutions to reduce the burden of performing several tasks during mobility.

Another important issue is the masking of sound, which is a situation generated through the overlap of various sounds. In most cities, background music emanates from various malls, department stores, underground shopping centers, and game centers. This type of sound is quite different from those that are emitted from crowds or vehicles. When these sounds are released simultaneously and when they are mixed with other noises, such as footsteps and traffic, they mask important signals or the sounds that a person who is visually impaired is trying to keep track of, as reported by Seki [19]. Other factors that contribute to these acoustical inconveniences include the echoes caused by buildings, constantly changing background noise, and the sounds of people moving in different directions, as stated in Figure 1.

Thus, we ought to pay attention to suggestions and new techniques for improving information dissemination via auditory means, as listed in Jonnalagedda et al. [5]. The authors list four items:

1. The modification to broadcasting systems and improving sound quality in public places;
2. The announcers and operators who operate these systems could benefit from additional training;
3. Paying special attention to acoustical issues such as sound echo when selecting and executing a building design when choosing construction materials;
4. The development of visual information systems by using magnified signs and high contrast to offer information in various forms.

One main issue that emerged from these findings is the need to develop effective methods of related spatial information using non-visual mediums. Alternative non-visual ways to convey information may happen through two possible methods: The first is the development of new systems utilizing haptic signs. The second is the modification and development of systems based on audible cues.

Tactile maps and Braille indication boards that convey information via haptic means have already been installed in many locations. Still, users who are blind cannot use them, since it is impossible to be aware of their locations, particularly in unfamiliar environments. Moreover, the number of Braille users among the visually impaired is still comparatively small. Therefore, modified auditory cues could provide the visually impaired access to detailed information that would support environmental learning, location familiarization, route selection, and decision-making.

The study concludes that audible pedestrian signals can be a useful device for providing additional information to standard visual pedestrian signals. Utilizing audible sound is not the only way to achieve efficient and independent travel; other strategies can also be of great benefit for developing orientation ability and to use other senses besides seeking assistance from other pedestrians.

### 4.1. Implication of the Acquired Results

A number of suggestions and new techniques can be inferred based on the data revealed by this study to be used when designing new building to improve information dissemination via audio means for people who have a visual impairment. For instance, the data revealed significant differences between participants from developed and developing countries in terms of the strategies that they use while wayfinding, with participants from developed countries mainly relying on designed auditory cues for the visually impaired. Thus, they can benefit from the installation of auditory cues in railway station facilities, turnstiles, and ticket offices. Other auditory cues can be installed at building entrances, exits, and elevators, and auditory cues can be installed to help distinguish between upward- and downward-moving escalators and the male and female toilets. Additionally, devices that provide on-demand minimal auditory feedback can be helpful to reduce unnecessary noise. However, participants from developing countries can benefit from several adjustments to help them when using new building structures. The following list presents examples of such adjustments: modifications to broadcasting systems and improving sound quality in public places; additional training for announcers and operators who operate these systems; and the importance of paying special attention to acoustics issues such as sound echo when selecting and executing a building design. Furthermore, choosing construction materials and the development of visual information systems using magnified signs and high levels of contrast could help to offer information in various forms.

### 4.2. Limitations

This study experienced challenges in terms of sampling and recruiting more participants than those who are included here in order to create a broader sample size. This was partially due to language barriers since interviews were only conducted in languages spoken by the interview or that could be translated by people within the interviewer’s network. Furthermore, having more diversity than is reflected in the study among the types of visual impairments would have strengthened the results. Another challenge was the time difference between Japan, where the study was conducted, and many of the interviewees’ countries of residence. In many cases, this made it difficult to set a convenient interview time for many who had initially expressed an interest in participating. As a result, the number of interviewees decreased considerably from the number that was originally expected.

Despite these limitations, the interview data were useful as a starting point for recommendations and for the design universal guidelines for auditory cues that can help the visually impaired achieve even greater independent mobility. Further research can more strengthen the results obtained by the current research, and thus, the findings can be widely generalized.

## 5. Conclusions

The study highlights various problems that are faced by people with a visual impairment during individual mobility and when using public transport systems. The main challenge appears to be maintaining an immediate and constant source of information to keep up-to-date with the surrounding environment. A constant source of information is deemed necessary to reduce the stress that the visually impaired experience when performing various tasks simultaneously. The participants expressed a need for auditory cues to be installed in railway station facilities, bus stops, public facilities, and food and beverage locations.

These findings, while still preliminary, may be the basis for developing and proposing context-based and specific actions that are suitable for systems that provide minimal audio feedback. Consequently, such actions may result in a number of benefits for commuters with visual impairment, such as reducing the masking of natural sounds. Indeed, audible assistance, such at airports and railway stations or train and bus announcements, are some of the most important sources of information for the visually impaired. However, they are temporary and may be considered an annoyance for other travelers. Special attention should be given to priority facilities where installing such devices is appropriate based on associated economic factors. Another factor that deserves attention when designing new sounds—as stated above in the Results section—is the use the pre-existing creative competencies of people who are visually impaired. For example, the pitch of audio signals should change to indicate the directions in which escalators and elevators are moving. Additionally, new sounds should be unique and easy to distinguish from other overlapping sounds.

When developing or installing new devices to disseminate information and to provide orientation, it is important that the intended user can optimize their use. Such an approach is deemed necessary if the visually impaired are to enjoy equal opportunity and to use public infrastructure to its full capacity.

## Figures and Tables

**Table 1:** Participants’ profile.

**Age**	
under 30	8
31 to 40	18
41 to 50	17
51 to 60	10
over 60	7
**Sex**	
male	35
female	25
**Disability type**	
totally blind	43
partially sighted	17
**Employment status**	
student	15
employed	35
unemployed	10

**Table 2:** Essential information required by the participants while navigating at an unfamiliar location.

Train Users	Grown City	Response	Other Transportation Systems’ Users	Grown City	Response
Exits and entrances locations and numbers	DC	17	Destination address	SDC	14
	SDC	2			
Stairs location	DC	19	Fuel stations	SDC	10
Escalators location	DC	23	Shops	DC	3
				SDC	10
Elevators location	DC	19	Well-known buildings	SDC	14
	SDC	3			
Toilets location	DC	20	Exact bus stop	DC	4
				SDC	15
Ticket vending machines location	DC	20	Taxi stations	DC	20
				SDC	30
Coach number	DC	16	Restaurants	SDC	21
	SDC	2			
Shopping areas	DC	15	Hotels	DC	15
				SDC	2
Reading information on boards and fare table	DC	15	ATM and bank locations	DC	16
				SDC	2
Name of transfer stations	DC	19	Public parks and recreation facilities	DC	1
	SDC			SDC	15
Distance between station and final destination	DC	22	Hospitals	DC	4
	SDC			SDC	9

DC: developed cities (Japan, England, South Korea, New Zealand, China, USA); 33 participants, SDC: slightly developed cities (Sudan, Egypt, Jordan, Thailand, Indonesia, Kyrgyzstan); 27 participants.

**Table 3:** Natural and artificial auditory cues as a source of information in unfamiliar environments.

Train Users	Grown City	Response	Other Transportation Systems’ Users	Grown City	Response
Beeps of cashier and bus electronic cards	DC	20	Sound of scissors at barber shop	SDC	7
	SDC	2			
Train station chime	DC	16	People selling items on the street	SDC	15
Artificial bird sound at stairs	DC	13	Mobile catering vehicles	SDC	4
Electronic announcement sound indicating escalators locations	DC	11	Deep-oil fryers	SDC	3
			Glass cups at coffee shops	DC	2
				SDC	16
			Wind sound at corners	SDC	12
			Sound of river water flow	SDC	10
			Animals and birds	DC	2
				SDC	12
			Cars passing speed bumps	DC	16
				SDC	11
			Car maintenance equipment	SDC	14
			People sounds at kindergartens, schools, playground	DC	20
				SDC	18

DC: developed cities (Japan, England, South Korea, New Zealand, China, USA); 33 participants, SDC: slightly developed cities (Sudan, Egypt, Jordan, Thailand, Indonesia, Kyrgyzstan); 27 participants.

**Table 4:** Hopes regarding installation of auditory cues at different locations.

Train Users	Grown City	Response	Other Transportation Systems’ Users	Grown City	Response
Station entrance and exits	DC	23	Taxi and bus stops	SDC	22
	SDC	3			
Distinguishing up and down escalators	DC	12	Hospitals and pharmacies	DC	2
				SDC	13
Male and female toilets	DC	15	Post offices and banks	DC	9
				SDC	5
In–out tickets gate	DC	20	Restaurants and coffee shops	SDC	22
Turnstiles	DC	19	Building entrances and public parks	DC	12
				SDC	3
Ticket offices	DC	20	Shopping malls and supermarkets	DC	23
				SDC	6
Elevators	DC	18	Vending machines and laundry areas	DC	18
				SDC	6

DC: developed cities (Japan, England, South Korea, New Zealand, China, USA); 33 participants, SDC: slightly developed cities (Sudan, Egypt, Jordan, Thailand, Indonesia, Kyrgyzstan); 27 participants.

## Data Availability

Data is contained within the article. Individual data more detailed than those in the article will not be made public due to privacy issues.
